# Vitamin B_6_ and Its Role in Cell Metabolism and Physiology

**DOI:** 10.3390/cells7070084

**Published:** 2018-07-22

**Authors:** Marcelina Parra, Seth Stahl, Hanjo Hellmann

**Affiliations:** Hellmann Lab, School of Biological Sciences, College of Liberal Arts and Sciences, Washington State University, Pullman, 99164-6234 WA, USA; marcelina.parra@wsu.edu (M.P.); seth.stahl@wsu.edu (S.S.)

**Keywords:** cell, health, metabolism, pathogen, PLP, physiology, pyridoxine, stress, vitamin B_6_

## Abstract

Vitamin B_6_ is one of the most central molecules in cells of living organisms. It is a critical co-factor for a diverse range of biochemical reactions that regulate basic cellular metabolism, which impact overall physiology. In the last several years, major progress has been accomplished on various aspects of vitamin B_6_ biology. Consequently, this review goes beyond the classical role of vitamin B_6_ as a cofactor to highlight new structural and regulatory information that further defines how the vitamin is synthesized and controlled in the cell. We also discuss broader applications of the vitamin related to human health, pathogen resistance, and abiotic stress tolerance. Overall, the information assembled shall provide helpful insight on top of what is currently known about the vitamin, along with addressing currently open questions in the field to highlight possible approaches vitamin B_6_ research may take in the future.

## 1. Introduction

Vitamin B_6_ (vitB_6_), or pyridoxine, is a very important compound for general cellular metabolism [1]. Since its discovery in 1934 by György and colleagues [2], it has been implicated as a co-factor in more than 140 biochemical reactions in the cell [3]. Although most vitB_6_ co-catalyzed reactions are related to amino acid biosynthesis and catabolism, vitB_6_ also contributes to fatty acid biosynthesis, breakdown of certain storage compounds in animals and plants, as well as in the biosynthesis of plant hormones, neurotransmitters, and organelle-specific compounds such as chlorophyll [4,5,6,7,8,9,10]. In addition, vitB_6_ can quench reactive oxygen species (ROS) [11]. Because of its role in ROS scavenging and chlorophyll synthesis, vitB_6_ is beneficial for photosynthesis, and is discussed as a possible factor to alleviate abiotic and biotic stress [11]. 

**Figure 1:**
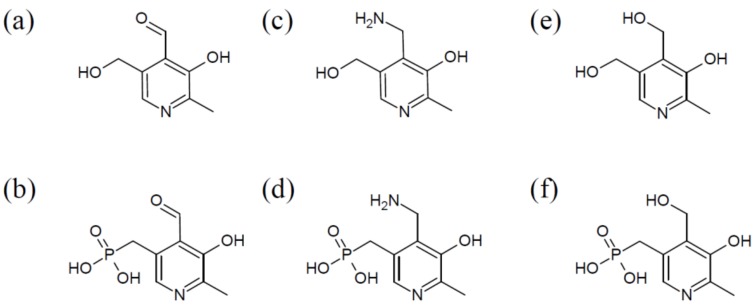
Chemical structures of the six common B_6_ vitamers. (**a**,**b**): Pyridoxal (**a**) and its phosphorylated form pyridoxal 5′-phosphate (**b**), (**c**,**d**): pyridoxamine (**c**) and pyridoxamine 5′-phosphate (**d**), (**e**,**f**): pyridoxine (**e**) and pyridoxine 5′-phosphate (**f**).

The vitamin comprises a group of six chemically related compounds that all contain a pyridine ring as their core. They differ from each other in a variable group at the pyridine’s 4′ position, which can either be an amino methyl group (pyridoxamine (PM)), a hydroxyl methyl group (pyridoxine (PN)), or an aldehyde (pyridoxal (PL)) (Figure 1). Once the different derivatives are phosphorylated, they can function as co-factors, with pyridoxal 5′-phosphate (PLP) being the biologically active form of vitB_6_.

In the following review, we will briefly summarize the known biosynthetic pathways in eu- and prokaryotes, and then focus on transport, cellular roles, potential regulatory steps that affect its rate of synthesis, and its importance in human dietary and health issues.

**Figure 2:**
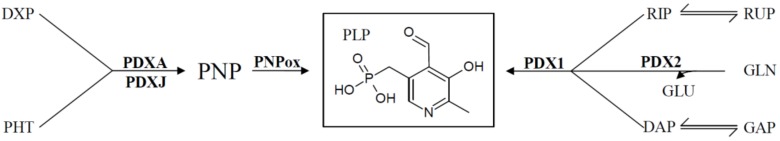
The two known de novo pathways leading to the biosynthesis of PLP. The two de novo pathways, DXP-dependent and -independent, use pyridoxine 5′-phosphate synthase (PDXJ) and 4-hydroxythreonine-4-phosphate dehydrogenase (A) and PDX1 and 2 enzyme, respectively, to synthesize PLP. DXP, Deoxyxylose 5′-phosphate; DAP, Dihydroxyacetone phosphate; DXP, 1-Deoxyxylulose-5-phosphate; GAP, Glyceraldehyde-3-phosphate; GLN, L-Glutamine; GLU, L-Glutamate; PHT, 4-Phosphohydroxy-L-threonine; PLP, Pyridoxal-5-phosphate; PNP, Pyridoxine-5-phosphate; RIP, Ribose-5-phosphate; RUP, Ribulose-5-phosphate.

For many years the only biosynthetic pathway known came from the prokaryote *Escherichia coli*. The pathway is comparably complex, but ultimately leads to the pyridoxine biosynthesis proteins A (PDXA; a 4-hydroxythreonine-4-phosphate dehydrogenase) and PDXJ (a pyridoxine 5′-phosphate synthase) that utilize deoxyxylose 5′-phosphate (DXP) and 4-phospohydroxy-L-threonine to synthesize pyridoxine 5′-phosphate (PNP). A flavin mononucleotide (FMN)-dependent PNP oxidase (PNPox) then converts PNP to PLP (Figure 2) [12,13,14]. An alternative pathway was only relatively recently discovered in fungi; plants; and some bacteria that uses a 3-carbon sugar (either glyceraldehyde 3′-phosphate or dihydroxyacetone phosphate), a pentose-phosphate (either ribose 5′-phosphate or ribulose 5′-phosphate), and glutamine to synthesize PLP [15,16]. Two critical enzymes called PDX1 and PDX2 form a larger PLP synthase complex to catalyze the formation of the vitamin [15,16,17,18]. This newer pathway is commonly referred to as the deoxyxylose 5′-phosphate (DXP)-independent pathway, to distinguish it from the biosynthetic pathway present in *E. coli* that depends on deoxyxylose 5′-phosphate as a precursor (Figure 2). Of note is that humans and most animals do not encode for any of the de novo enzymes, but rely solely on external supplies of the vitamin from their food. As such, vitB_6_ is an essential nutrient to humans.

The importance of the de novo pathway is emphasized by various studies in *E. coli*, *Streptococcus pneumonia*, *Bacillus subtilis*, yeast, and plants where its complete loss is lethal to the organism, but can be rescued by an exogenous supply of the vitamin [17,18,19,20,21,22,23,24]. Even a reduction in the biosynthetic efficiency causes severe developmental problems. For example, the plant *Arabidopsis thaliana* has two functional homologs of PDX1 involved in the de novo biosynthesis of vitB_6_ [16,18]. Both PDX1 proteins are expressed throughout the plant and appear to be mostly functionally redundant [18,19,25]. However, *Arabidopsis* null mutants affected in either one of the two PDX1 proteins show severe developmental defects, including stunted root growth, smaller rosette leaves, delayed flowering [17,18], and considerably larger cells, compared with wild-type [26]. Plants also developed larger seeds with increased protein, lipid, and carbohydrate contents [26].

**Figure 3:**
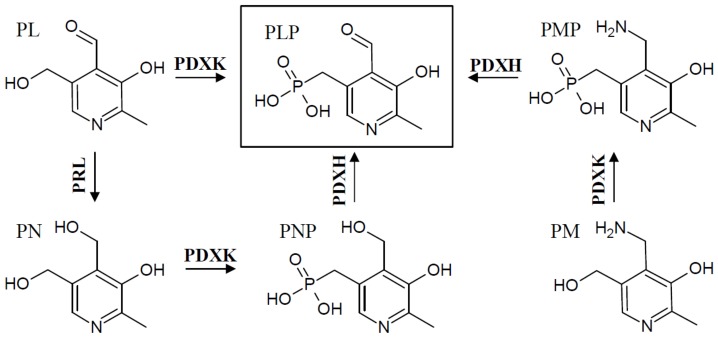
The VitB_6_ salvage pathway. The salvage pathway is used to derivatize and phosphorylate B_6_ vitamers, leading to PLP synthesis via oxidase (PDXH), kinase (K), and pyridoxal reductase (PRL) enzymes.

In addition to the two known de novo pathways, most, if not all, organisms have a salvage pathway that is able to convert and phosphorylate the different B_6_ vitamin derivatives into the catalytically active form, PLP. This is accomplished by the combined activities of at least three enzymes, an oxidase (PDXH), a pyridoxal reductase (PRL), and a kinase (PDXK) (Figure 3) [27,28,29,30]. At least two different kinases have been described that are either specific for PL (further referred to as PLK), or which recognize PN, PM, and PL as substrates (further referred to as PDXK), to generate the phosphorylated B_6_ vitamers [28,29,31]. The oxidase catalyzes the conversion of PNP and PMP to PLP [32,33], and the reductase forms PN from PL [34].

The salvage pathway is critical to make any non-phosphorylated B_6_ vitamers available to the cell as co-factors. This is generally relevant for PL, PM, or PN taken up with food and for their recycling in the cell. Consequently, it is essential for animals that depend on the pathway to interconvert the different B_6_ vitamers into PLP and to make them available as co-factors in enzymatic reactions. In addition, cellular phosphatases have been described that can de-phosphorylate PLP [35]. This may be needed to control the homeostasis of PLP in the cell, and can allow PL catabolism into 4-pyridoxic acid by an aldehyde oxidase [36].

In the liver of mammals, PLP becomes tightly bound to serum albumin as a Schiff base before being secreted into the circulatory blood system for delivery to the different tissues and organs [37,38]. Binding to albumin is discussed as a protective step against early de-phosphorylation of the vitamin. However, before uptake into the cell, PLP needs to be de-phosphorylated to PL by an extracellular, tissue-nonspecific alkaline phosphatase (ALP) [39,40,41]. As ALP only uses free PLP as a substrate, the uptake of PL is likely dependent on the rate PLP dissociates from albumin in the circulatory system.

The salvage pathway is without a doubt vital for animals. Just to provide one example, a genetic study in which the PDXH enzyme was knocked-out via RNAi in *Drosophila* led to flies that could not survive without additional supplementation of PLP [42]. Even PN was not able to compensate for the loss of the oxidase, corroborating that PLP is indispensable for cell functioning [42]. We are not aware of any described *pdxK* null mutants in animals (including human), but one can expect that such a mutant would also require a specific PLP-enriched diet to survive.

In comparison to animals, one would assume that organisms that have a de novo pathway do not require the salvage pathway for their survival. In fact, the few described examples show only mild developmental defects, but with a general tendency towards increased stress sensitivity in the affected organisms. For example, in the yeast *Saccharomyces cerevisiae*, loss of PDXH leads to increased sensitivity to oxidative stress caused by hydrogen peroxide [43]. Likewise, loss of either PDXK or PDXH also results in enhanced stress sensitive phenotypes in Arabidopsis plants [27,44]. Plant mutants affected in PDXH accumulate PNP and PMP with a decrease in PLP, which in turn may also limit PLP-dependent reactions [45]. Surprisingly, Arabidopsis *pdxK* mutants had nearly nine-fold elevated PLP levels compared with wild type [27]. Because this correlated with a strong up-regulation of the de novo pathway, it suggests that the de novo and the salvage pathways are tightly co-regulated [27]. Interestingly, *pdxH* mutants also showed mild aberrant developmental features, such as curled leaves or changed flowering time [45], but this was only observed in the absence of ammonium as the nitrogen source. The ammonium-dependency correlated with strongly reduced nitrate reductase (NR) activity in the cells of *pdxH* mutant plants. NR is required for the conversion of nitrate to ammonium, but may also be inhibited by PMP, as discussed by Colinas and co-workers [45]. Consequently, ammonium requirements in *pdxH* mutants may be the result of high PMP levels that negatively affect NR activity [45]. Of note is also that in fungi and plants, the salvage pathway might be critical for providing the organelles with a sufficient supply of PLP. While the de novo biosynthetic enzymes, as far as it has been described, are located in the cytoplasm, there is good evidence that salvage pathway enzyme are present in the cytosol and the organelles [43,44,46,47].

## 2. Complex Organization of PLP Synthases

The PDX1/PDX2 protein pair, which forms a PLP synthase, is widely distributed among archaea, bacteria, fungi, protists, and plants, and the corresponding complexes have been characterized in a variety of pro- and eukaryotes, including *Geobacillus stearothermophilus* (PdxS/PdxT) [48,49], *B. subtilis* (Pdx1/Pdx2 or PdxS/PdxT) [50,51], *Thermotoga maritima* (YaaD/YaaE) [52], *S. cerevisiae* [52,53], *A. thaliana* [54], and *Plasmodium falciparum* [55].

In all of these organisms, the PLP synthase is composed of 12 PDX1 units, and up to 12 PDX2 units. PDX1 proteins form two hexameric rings that interlock into a dodecamer. The hexamers align with each other in a way that a PDX1 protein on one hexamer interacts with two PDX1 molecules from the other hexamer [49,50,55]. This dodecameric double ring system is stable and does not require any specific substrate for PLP synthesis to retain its complex formation [50,55]. In contrast, PDX2s interact with the PDX1 ring only transiently, and this interaction requires the presence of glutamine [50,55]. After the amino group has been removed from glutamine by hydrolysis, the PDX2 proteins are more likely to leave the complex if no other glutamine is readily available for hydrolysis [55].

In plants, two recent studies have shown that PLP synthases contain a *lysine swing* or *lysine relay* mechanism that allows intermediate channeling of the substrate in the process of vitB_6_ biosynthesis [54,56]. Essentially, the lysine residues anchor the substrate to the PLP synthase and facilitate transfer of the substrate between the two active sites efficiently without extra domains or coenzymes [54]. These two sites are present in PDX1 proteins and are designated as P1 and P2 [56]. A lysine residue in the P1 site covalently binds to the pentose phosphate. The pentose phosphate is dephosphorylated, and together with water and ammonia from glutamine, form a chromophoric I_320_ intermediate with an absorbance maximum at 320 nm. This is shuttled to the P2 site, where it is condensed with a 3-carbon sugar to yield PLP [54,56].

The reaction process of the PLP synthase using the relay mechanism is exciting because, without additional co-enzymes or co-factors, it can perform such a variety of biochemical reactions ranging from isomerization to imine formation, ammonia addition, aldol-type condensation, cyclization, and aromatization [56]. Though both of these studies were conducted in *Arabidopsis*, this type of relay mechanism is likely conserved among PLP synthases from different organisms, considering the close homology and structural similarity of PDX proteins.

Whether the PLP synthase activity is regulated in response to cellular demands or other environmental or developmental factors is currently not well understood. Two interesting examples, however, come from plants that shed some light on possible regulatory mechanisms. First, *A. thaliana*, and most likely other plants as well, have a PDX1 protein (in *Arabidopsis*, this is named PDX1.2), which is not directly involved in the biosynthesis of vitB_6_ [57]. In recent years, the function of PDX1.2 has been explored, with data indicating that PDX1.2 may serve as a regulator of vitB_6_ biosynthesis under abiotic stress. PDX1.2 is able to interact with itself and the other PDX1 proteins, but it does not interact with PDX2 [18]. The protein is minimally expressed under normal growth conditions, but certain abiotic stressors, such as heat, strongly induce its expression [27,57,58,59], correlating with an increase in vitB_6_ biosynthesis [57]. This up-regulation of vitB_6_ biosynthesis under stress is missing in mutants with reduced *PDX1.2* expression, making it overall likely that PDX1.2 acts as a positive regulator of PLP synthase activity in plants [57]. PDX1.2 may also have other functions independent of vitB_6_ because corresponding null mutants, in which the other PDX1 and 2 proteins are functional, develop an embryo lethal phenotype [58]. However, the precise reason for this developmental defect in these mutants still needs to be resolved.

The second example of a possible regulatory mechanism controlling PLP synthase activity is related to an acetolactate synthase (ALS), which has been found to interact with an *Arabidopsis* PDX1 protein in yeast-2-hybrid experiments [60]. ALS is the first enzyme in the branched-chain amino acid (BCAA) synthesis pathway, which leads to the production of leucine, isoleucine, and valine, for example. VitB_6_ is an essential co-factor for branched-chain amino acid transaminase (BCAT), the last step of BCAA synthesis [61]. Given that the results of the yeast-2-hybrid approach can be verified in planta, this finding could represent a novel connection between BCAA and vitB_6_ biosynthesis [60]. As BCAAs are not only synthesized in plants, but also in various other organisms including fungi, bacteria, and archaea, this potential regulatory interplay may have broader implications. Indeed, YggS, a PLP-binding protein that is widely distributed throughout bacteria, fungi, and eukarya, has been experimentally linked to BCAAs [62]. In an *E. coli yggS* knockout strain, excess PN led to a toxicity ring (a lack of cell growth on the media in a circle around the PN treatment), while supplementation of either PL or the BCAAs leucine and isoleucine suppressed the PN toxicity [62]. It is suggested that the suppression of PN toxicity by the two BCAAs bypasses the *yggS* knockout strain’s need for specific PLP-dependent enzymes, such as transaminase B, which catalyzes the last step in leucine and isoleucine synthesis [62].

It would be of interest to know whether PDX1 proteins undergo broader interactions with other PLP-dependent enzymes such as ALS. This may indicate a potential cellular feedback mechanism to either regulate vitB_6_ demand or for the production of certain amino acids.

## 3. Regulation of Salvage Pathway Genes

Regulation of the salvage pathway is critical to maintain PLP homeostasis, especially for organisms that lack the de novo biosynthetic pathway. In the following section, some of the known regulatory impacts that occur on the transcriptional and post-translational level are described.

An interesting example of transcriptional regulation comes from *Salmonella typhimurium*, a pathogenic Gram-negative bacterium that is mainly known for causing typhoid fever [63]. Here, PtsJ, a member of the MocR family of transcription factors, negatively regulates both *PdxK* and its own expression by binding to the respective promoters [63]. PtsJ contains a helix-turn-helix (HTH) motif for DNA binding at its N-terminal region, while its C-terminal region is related to the type I family of PLP-dependent enzymes, classically represented by aspartate aminotransferases [63,64]. Intriguingly, PtsJ can bind PLP, and this binding further enhances its repressor role in *S. typhimurium,* providing an elegant mechanism to control *PdxK* expression in a PLP-dependent manner [63]. Loss of PtsJ strongly up-regulates *PdxK* expression, but only mildly impacts *PdxH* [63]. In addition, PtsJ does not bind to the *PdxH* promoter, suggesting that other mechanisms are in place to control PdxH levels in *S. typhimurium* [63].

In *Bombyx mori* caterpillars, PLP levels are discussed to be critical for progression of the different larval stages, and recent research indicates that expression of *PDXH* and *PDXK* genes is under the control of the juvenile and molting hormones [65].

In *Arabidopsis*, one can observe that mutants lacking PDXH, PDXK, or PLR expression of the remaining salvage pathway genes is strongly up-regulated [27,34]. As loss of PDXK also results in higher PLP and total vitB_6_ levels, the up-regulation of PDXH in a *pdxK* mutant background is clearly not related to a vitB_6_ deficiency [34]. In addition, *PDXK* expression is repressed in roots upon salt stress treatment [44]. *PDXH* expression is widely inducible by light, heat, and different phytohormones, namely jasmonic acid, ethylene, and abscisic acid treatments. Similar to *PDXK,* salt treatment results in a down regulation of *PDXH*, as does exposure to drought [66].

An interesting example of transcriptional regulation comes from the fungal plant pathogen *Rhizoctonia solani* [67]. Here, a *PLR* gene was highly inducible by oxidative stress caused by paraquat and phenyl acetic acid treatments, and mildly responsive to hydrogen peroxide exposure [67]. Plants generate hydrogen peroxide as an early defense response upon fungal infections [68,69]. The fungal response pattern resembles that of classical reactive oxygen species (ROS) detoxification enzymes, such as catalase and glutathione-S-transferase [67]. Because de novo vitB_6_ genes were also up-regulated in the fungus, it is likely that this increase in vitB_6_ production contributes to quenching of ROS to protect the cell against their harmful impacts, further supporting the fungal effort to infect the plant tissue.

On the protein level, some factors have been described in *E. coli* and humans that regulate the activities of PDXK and PDXH. PDXK functions as a homodimer and requires mono- and bivalent metal ions such as K^+^ and Mg^2+^, respectively, as co-factors [29,70,71,72,73]. For the human PDXK, it has also been shown that the enzyme activity is highly stimulated by Na^+^, while Zn^+^ ions at a physiological pH of 7.3 had an inhibitory impact [70]. As K^+^ is the more prevalent ion under normal physiological conditions in the cell, it is likely that human PDXK acts in a Mg^2+^/K^+^ form [70]. Besides Zn^+^ ions, the activity of PDXK is also inhibited by MgATP. It is discussed that MgATP forms a ternary complex together with PLP at the active site, which reduces PDXK activity [30,74]. PDXH also functions as a homodimer and each subunit binds a single FMN as a co-factor [33,75]. The enzyme is inhibited by its product, PLP, as well as its substrate, PNP, while PMP does not affect its activity [76,77,78,79].

Because of the conserved nature of PDXK and PDXH proteins, one can expect that factors affecting their activities such as ions, PLP, or MgATP are likely very similar in most organisms.

## 4. Regulation of De Novo VitB_6_ Synthesis Genes

As indicated above, regulatory steps that directly affect the activity of de novo enzymes involved in vitB_6_ biosynthesis are not well defined. However, a variety of factors are known that control the expression of genes involved in the de novo pathways. These are mainly related to the overall growth situation of the respective organism, but in many cases, abiotic or biotic stress conditions also cause increased cellular gene expression levels of vitB_6_ biosynthetic genes. In the following section, we will provide a few examples of conditions regulating gene expression in the two pathways for pro- and eukaryotes.

In *E. coli*, expression of the *PdxA* gene is positively regulated when growth rates increase, and this up-regulation depends on Fis, a protein that can interact with and bend DNA, and is known to function as a transcriptional regulator [80,81,82]. Similarly, de novo vitB_6_ biosynthesis genes in *B. subtilis* (here called *YaaD* and *E*) and the yeast *Schizosaccharomyces pombe* have been reported to be positively regulated with increasing growth rates [23,83]. In *B. subtilis*, addition of PN and PL to the growth medium did not repress their expression, indicating that vitB_6_ is likely not causing a feedback regulatory loop on the transcriptional level [23].

In several prokaryotes that possess the DXP-dependent de novo biosynthesis pathway, such as *Bacillus caucillus*, *Listeria monocytogenes*, *Corynebacterium glutamicum*, and *S. pneumoniae*, it has been shown that expression of the genes encoding for the PLP synthase depends on PdxR [21,22,84,85]. PdxR is a MocR-related transcription factor that promotes expression of the de novo biosynthesis genes [86]. Like PtsJ, it has an HTH-DNA binding domain and a type I domain for PLP-dependent enzymes [86]. Its activity is down-regulated by binding to PLP, thereby providing, as described for *S. typhimurium,* a product-based feedback mechanism to control PLP biosynthesis on the transcriptional level in the cell [84].

Previous research has shown that vitB_6_ affects enzyme induction by steroid hormones [87]. These steroid hormone receptors belong to a superfamily of transcription factors that regulate physiological processes such as growth, development, reproduction, and behavior [88]. Work done by Cake et al. (1978) displayed that PLP could inhibit the binding to DNA-cellulose of the rat-liver glucocorticoid receptor, a receptor to which cortisol and other glucocorticoids bind [88,89]. This receptor was also found in humans and is similarly inhibited by PLP; however, it was also observed that estrogen and androgen receptors were also affected [88]. It is believed that vitB_6_ modulates the activities of different hormone steroid receptors on the transcriptional level [88]. For this binding to occur on nuclei, chromatin, or DNA, the receptor must undergo “activation” by a physiochemical alteration such as heat or ionic irregularities [89]. It has also been seen that enhanced levels of PLP can decrease the transcriptional response to these receptors, and deficiencies in vitB_6_ lead to an enhanced responsiveness to steroid hormones [87,88,89].

An interesting regulatory example comes from the bacterium *Photorhabdus luminescens*, which can switch between two lifestyles; a mutualistic (M) one, where it is in a symbiotic relationship with the nematode *Heterorhabditis bacteriophora*, and a parasitic (P) one, where it can infect other nematodes like *Caenorhabditis elegans*. Inversion of a single promoter controls whether *P. luminescens* assumes either the M or P state, which is followed by metabolic alterations and morphological changes, respectively [90]. As one can also observe different expression levels of *P. luminescens’ pdxA* and *J* genes in the M versus the P form (with a ratio of 2.1 for *pdxA*, and 0.49 for *pdxJ*, of M to P), the rate of vitB_6_ biosynthesis is discussed as a potential requirement for the bacterium to adopt the respective lifestyle [91].

A connection to abiotic stress can be found in *R. solani*, a plant pathogenic fungus, where transcript levels of *PDX1* and *PDX2* increase significantly when exposed to the superoxide generator paraquat [67]. Increased expression of *PDX* genes in response to abiotic stress, especially to conditions that increase ROS, has also been reported in other fungi, *B. subtilis*, and plants, and appears to be of a general nature [25,83,92,93,94,95,96]. These studies provide some evidence that PDXs are not only key proteins necessary for vitB_6_ biosynthesis, but are also vital factors for abiotic stress tolerance. This notion is corroborated, for example, by *pdx1 Arabidopsis* plant mutants that are hypersensitive to osmotic and oxidative stress [25]. In fact, *Arabidopsis PDX* genes are up-regulated by a variety of abiotic stress conditions such as high light, chilling, and drought [59]. The promoters of *PDX1* genes further contain a wide set of *cis*-regulatory elements that are recognized by MYB, MYC, and WRKY transcription factor families, and that are often involved in gene regulation after stress response [97,98,99,100]. *PDX1* promoters also contain abscisic acid-responsive elements (ABRE) and ethylene-responsive elements (ERE) [19]. Ethylene and abscisic acid are two classical stress-related phytohormones. In addition, a sugar response elements (SRE) was reported in PDX1 promoters [19], which is also of interest as one of the first plant PDX1 proteins identified came out of a screen for sugar-responsive mutants [18,101]. Although much of these data are based on in silico analysis, the variety of elements detectable in *PDX* promoters indicates that in plants and other organisms, PLP synthase activity is tightly intertwined with the general cellular metabolism and physiology of the cell.

## 5. Transport and Distribution of VitB_6_

**Figure 4:**
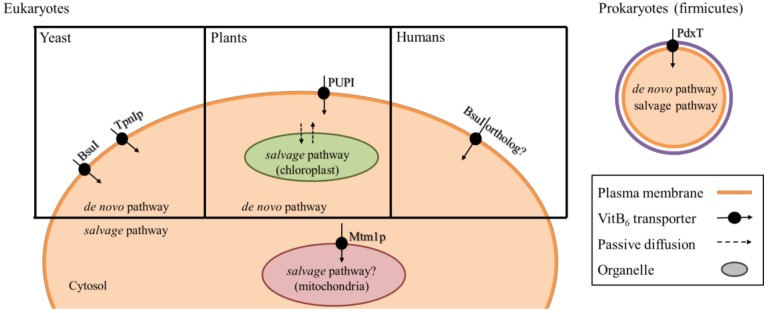
Schematic of known and potential vitB_6_ transporters**.** In the yeasts *S. cerevisiae* and *S. pombe*, import of vitB_6_ across the plasma membrane is facilitated by Tpn1p and Bsu1, respectively. In plants, members of the amino acid permease family (here PUP1) perform this function. In humans, there is no confirmed transporter for vitB_6_, but this may be accomplished by BsuI orthologs. In the bacterial phylum firmicutes, a modular transporter called PdxT transports B_6_ vitamers into the cell. Mtm1p is a carrier from *S. cerevisiae* that imports PLP into the mitochondria. De novo and *salvage* pathways are labeled according to their presumed subcellular localization and presence in the respective organism.

For all organisms, it is crucial to have sufficient vitB_6_ available in the cell. For organisms that lack a de novo biosynthesis pathway, it is further essential that they have an efficient extracellular uptake system in place. However, there are very few known transporters located at the plasma membrane, and even fewer known transporters that are present at the membranes of intracellular compartments [102]. Surprisingly, transporters for vitB_6_ in eukaryotes have only been described in yeast and plants that already have a de novo pathwfay (Figure 4). In the yeasts *S. cerevisiae* and *S. pombe,* two transporters called Tpn1p and Bsu1, respectively, facilitate import of PN, PL, and PM across the plasma membrane [103,104]. Tpn1p belongs to the purine cytosine permease family, and its activity is up-regulated when vitB_6_ levels decrease in the yeast growth medium [104], although the mechanism of this regulation remains open. Bsu1 shows similarity to the MULTIDRUG RESISTANCE (MDR) family of transport proteins [103]. Of note is that it also facilitates transport of the antihypertensitive drug amiloride that inhibits Na^+^/K^+^ transporters [105]. Because amiloride is an effective drug to treat patients that suffer from low levels of K^+^ in the blood serum (hypokalemia) [105,106,107], it is discussed that an unknown ortholog of BsuI may facilitate vitB_6_ uptake in humans (Figure 4) [103,108].

Recently, members of the plant purine permease (PUP) family, who were originally identified as transporters for the phytohormone cytokinin, also showed the ability to transport PN, PL, and PM [109,110,111]. Work in *A. thaliana* on PUP1 showed that only non-phosphorylated vitB_6_ derivatives are transported, not the active PLP form [111]. Work in tobacco on the PUP member NICOTINE UPTAKE PERMEASE 1 (NUP1) yielded similar results [110]. Interestingly, NUP1 was originally described to facilitate transport of nicotine from the apoplastic space, and to affect alkaloid metabolism. As such, the vitB_6_ transport added a new and interesting facet to NUP1 function in tobacco. Overall, the precise meaning of plant purine permeases transporting B_6_ vitamers is not fully resolved. One discussed aspect is that they may allow long-distance distribution of non-phosphorylated B_6_ vitamers to supply vitB_6_ taken up by the roots from the soil to other organs [110,111]. Nevertheless, it is unclear to what extent such transport is indeed a critical requirement for plants, as the genes encoding for the DXP-independent pathway are expressed in all tissues [18]. In addition, as outlined above, these PUPs have broad substrate specificity, and are thus likely to be required in the cell for a much wider range of physiological processes than just vitB_6_ metabolism.

The majority of known vitB_6_ transporters from yeast and plants are likely located at the plasma membrane (Figure 4). This leaves the question open on how the organelles are supplied with the vitamin. Subcellular localization studies of PDX1 and PDX2 proteins in plants showed that the de novo synthesis via the DXP-independent route most likely takes place only in the cytosol [16]. This is to some degree surprising, as major steps in vitB_6_-dependent amino acid metabolism are present in mitochondria and chloroplasts [46,112]. It was found that in *S. cerevisiae*, the mitochondrial carrier protein Mtm1p is a high affinity importer that is responsible for transport of PLP across the mitochondrial membrane [113]. Interestingly, although Mtm1p is not an iron carrier, it plays a role in mitochondrial Fe–S cluster biosynthesis, and its deletion results in defects in iron homeostasis and heme biosynthesis, highlighting a link between PLP transport and mitochondrial iron homeostasis [113]. In plants, it remains ambiguous how chloroplasts are sufficiently supplied with the vitamin, especially because the end-product of the DXP-independent pathway is PLP, a charged compound that cannot passively diffuse across the membrane, and that is not transported by PUPs [111].

As mentioned above, in contrast to the de novo pathway, the salvage pathway in plants, and likely other eukaryotes, appears to take place in the cytosol, as well as in the organelles. At least for PDXH and PDXK from *Arabidopsis*, localization in the chloroplasts has been reported (Figure 4) [43,47]. If the non-phosphorylated forms of the vitamin can passively diffuse into the chloroplast, the presence of the salvage pathway in the organelle may assure availability of PLP for enzymatic reactions. However, it remains questionable whether a passive system would supply sufficient amounts to satisfy the vitB_6_ demand in the chloroplast.

Alternatively, the organelles may have yet undescribed vitB_6_ transporters of their own that differ from the ones present at the plasma membrane. One possible example relevant for eukaryotes comes from the bacterial phylum firmicutes, where a modular built transporter, PdxT, of the ATP BINDING CASSETTE (ABC) family, can transport vitB_6_ (Figure 4) [114]. The transporter is coupled with a PdxK, likely to directly phosphorylate and activate B_6_ vitamers after their uptake into the cell [114].

In contrast to the biosynthetic pathways, knowledge explaining transport over long distances and within the cell is currently only poorly understood in eukaryotes and will require more thorough investigations to fully comprehend vitB_6_ metabolism in the cell.

## 6. Vitamin B_6_ and Its Involvement in Cellular Metabolism

As mentioned in the beginning, vitB_6_ is involved in more than 140 different metabolic reactions in the cell. To provide detailed information about all its functions would exceed the aim of this review. Therefore, in the following section, we will depict a few examples in which PLP has a very crucial role in cellular metabolism and physiology to provide a better appreciation of this vitamin’s role in the cell.

### 6.1. VitB_6_ Involvement in Protein Folding

PLP has also been reported to play a role as a chaperone in folding of PLP-enzymes [115]. For instance, cystalysin, a hemolytic protein from *Treponema denticola*, requires PLP for proper folding and stabilization of the protein [115,116]. The aspartate aminotransferase in *E. coli* requires PLP for proper stabilization in a native structure, and as the protein disassociates into a partially folded intermediate, PLP is reduced into a pyridoxyllysine (PPL) derivative to ‘lock’ the co-factor in place to further stabilize the protein [117]. Found in *E. coli* and *B. subtilis* as well, serine hydroxymethyltransferase (SHMT), which is part of the folate cycle, does not require PLP for folding, but is still required for proper functioning of the protein [115,118]. There is very little known about the function of vitB_6_ as a chaperone, thus more research is required, although perhaps the lack of research available is indicative of a very limited role.

### 6.2. VitB_6_ Involvement in Amino Acid Biosynthesis

Probably the most important function of active, phosphorylated vitB_6_ in the cell is related to the biosynthesis, as well as the degradation of amino acids. Here, the vitamin is often used for transamination reactions where, by forming an amino acid–PLP Schiff base intermediate, an amino group is transferred to a ketoacid to form new amino acids. In addition, vitB_6_ is also used for other types of reactions such as α-decarboxylations or racemizations [119].

The impact on amino acid metabolism is exemplified in plants overexpressing a PDX1 protein. Here, significantly increased levels of methionine, glycine, and proline were detected, while there was a decrease in not only γ-amino butyric acid and asparagine, but also in carbohydrates, such as glucose and sucrose [26,120]. In comparison, PDX2 overexpressing plants had decreased levels of β-alanine, arginine, and glutamine, however, there was an increase in proline levels [120]. In plants overexpressing both *PDX1.1* and *PDX2*, a more general, strong increase in sugars and amino acids was detected, along with a decrease in organic acids [26], indicating that an even up-regulation of both PDX1 and PDX2 is a requirement to promote amino acid biosynthesis on a broader scale.

The function of PLP-dependent enzymes in amino acid metabolism also has significant indirect impacts on secondary metabolites that depend on amino acids as their precursors. For example, this is the case for some plant hormones (phytohormones) such as ethylene, auxin, or cytokinin [5,121,122,123]. Auxin biosynthesis requires a tryptophan aminotransferase that catalyzes the step to indole-3-pyruvate, an immediate precursor of the auxin indole-3-acetic acid from L-tryptophan and 2-oxoglutarate [122]. Ethylene biosynthesis depends on the PLP-dependent enzyme ACC (1-aminocyclopropane-1-carboxilic acid) synthase, which generates ACC from S-Adenosyl methionine [5,123], and cytokinin biosynthesis involves lysine decarboxylases, which are known to need PLP as a co-factor [123].

Consequently, it has been demonstrated for auxin and ethylene that homeostasis of these two phytohormones is affected in plant mutants with strongly reduced abilities to synthesize vitB_6_ [19]. Because auxin is critical for root development, these findings correspond to the shorter root growth seen in vitB_6_ deficient plants [19].

### 6.3. VitB6 and Degradation of Cellular Storage Compounds

Glycogen and starch are two major storage compounds in animals, fungi, bacteria, and plants. Glycogen is a branched polymer that mainly consists of α-1,4-glycosidic bonds with every tenth glucose moiety also forming α-1,6-glycosidic bonds. It is required in humans as an energy storage form in brain and muscle cells that can be quickly made available [124]. Starch is a major carbohydrate storage form in plastids, and is also built up by α-1,4- and α-1,6-glycosidic bonds [125]. Glycogen can be made accessible to the cell by glycogen or starch phosphorylase, a PLP-dependent enzyme that hydrolyzes the α-1,4-glycosidic bonds to generate glucose-1-phosphate [81,83,124,126]. In mammals, glycogen phosphorylase is found predominantly in the liver, muscle, and brain [127]. Inhibition of glycogen phosphorylase is discussed as a way to control the glycemic blood sugar levels in affected patients, and a possible mechanism to treat type 2 diabetes [128,129].

### 6.4. VitB6 and Its Relevance in Tetrapyrrole Biosynthesis

Hemes, cobalamins, and chlorophylls are all tetrapyrroles required for cellular metabolism in animals, plants, and microorganisms, respectively. Heme is an iron-binding prosthetic group in metalloproteins, likely best known for its function in hemoglobins to bind oxygen for long distance transport in erythrocytes [130]. Cobalamin (vitB_12_) is a cobalt-binding molecule that is ubiquitously required in living organisms for fatty acid and amino acid metabolism, as well as DNA synthesis [131]. Chlorophyll is a magnesium-binding porphyrin that is essential for photosynthesis in plants, algae, and cyanobacteria [132,133]. Biosynthesis of all of these compounds depends on the activity of aminolevulinic acid synthase, a PLP-dependent enzyme that uses succinyl–CoA and glycine to generate δ-aminolevulinic acid, an immediate precursor in tetrapyrrole biosynthesis [134,135].

### 6.5. VitB_6_ and Its Role in Neurotransmitter Biosynthesis

**Figure 5:**
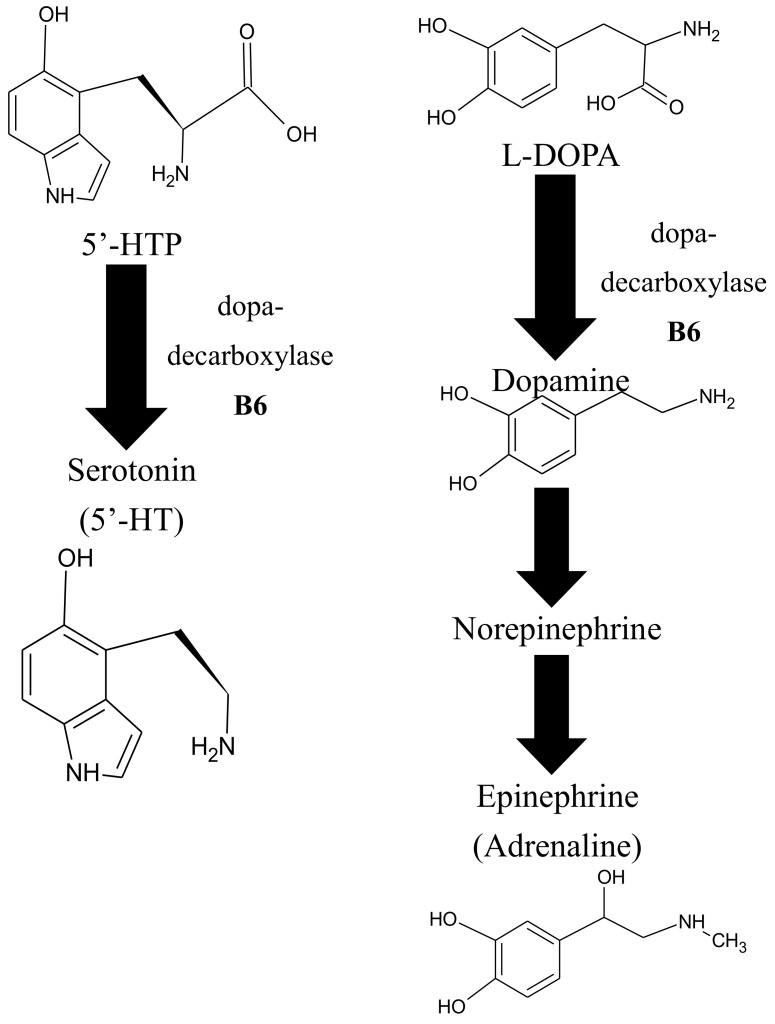
Schematic drawing of neurotransmitter biosynthesis that involve PLP-dependent enzymes. B6, vitB_6_; L-DOPA, L-3′-4′-dihydroxyphenylalanine; 5′-HTP, 5′-hydroxytryptophan; 5′-HT, 5′-hydroxytryptamine.

Epidemiological surveys have found that a greater intake of foods that contain high amounts of vitB_6_ correlates with better mental health [136,137,138,139,140]. This may be related to the fact that vitB_6_-dependent enzymes are needed for the biosynthesis of at least three important neurotransmitters; epinephrine (also known as adrenaline), dopamine (dihydroxyphenethylamine), and serotonin [138] (Figure 5). Epinephrine is critical for acute stress responses (*fight-or-flight response*) in humans [141] and is classically used as a medication for anaphylaxis, a severe allergic reaction, or cardiac arrest [142,143]. Dopamine can be considered a ‘reward’ neurotransmitter that can regulate positive emotions but also motoric control, and it has been associated with Parkinson’s disease and schizophrenia [144,145,146,147,148]. Serotonin (or 5-hydroxytryptamine) is known as a neurotransmitter that helps patients with depression by promoting well-being and contentment [149,150,151]. In this context, it is of note that depression is predicted to be the number one cause of mental disorders by the World Health Organization by the year 2030. It has become a public health problem here in the United States [152], and multiple studies have shown that deficiencies in vitB_6_ negatively affect mental health and cognitive abilities [138,140,153,154].

The biosynthesis of epinephrine, serotonin, and dopamine is independently influenced by a universal methyl donor, S-adenosylmethionine (SAM), a critical intermediate in the methionine-cycle [140,155,156,157,158,159,160] (Figure 5). Consequently, several studies have shown that a deficiency in vitB_6_ correlates with a deficiency in folate and cobalamin [138,140,153,154].

**Figure 6:**
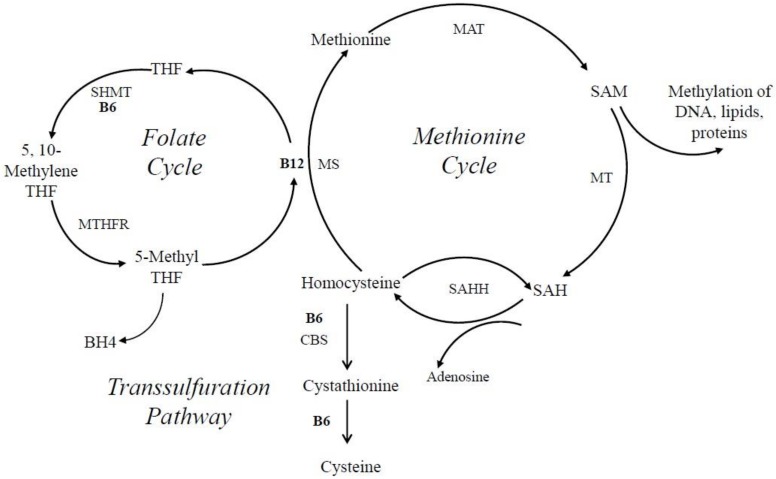
Schematic drawing of the 1-carbon methylation cycle. The methionine and the folate cycle, as well as the transulfuration pathway, are three interrelated routes involved in one-carbon metabolism, which is required for the synthesis of amino acids, neurotransmitters, and methylation of DNA and proteins. B6, vitB_6_; B12, vitB_12_; BH4, tetrahydrobiopterin; CBS, cystathionine-β-synthase; MAT, methionine adenosyltransferase; MS, methionine synthase; MT, methyltransferase; MTHFR, methylenetetrahydrofolate reductase; SAH, S-adenosylhomocysteine; SAHH, S-adenosylhomocysteine hydrolase; SAM, S-adenosylmethionine; SHMT, serine hydroxymethyltransferase; THF, tetrahydrofolate.

Homocysteine is methylated to methionine by 5-methyltetrahydrofolate, which is needed for the production of purines and thymidylate. Methylation of homocyteine to methionone depends on two other important vitamins, cobalamin (vitB_12_), and folate (vitB_9_), where PLP-dependent enzymes such as SHMT, which converts tetrahydrofolate (THF) to 5, 10-methylene tetrahydrofolate, are involved (Figure 6). Elevated levels of homocysteine, otherwise known as hyperhomocysteinemia, are recognized as a cardiovascular risk factor; are a predictor of primary-cause vascular mortality; and have also been associated with mental retardation, seizures, depression, schizophrenia, and cognitive impairment [138,153,160,161]. Homocysteine can be converted in three different ways in the cell. One possibility is the conversion to cysteine via a transulfuration pathway by the PLP-dependent enzyme cystathionine β-lyase. Alternatively, it can be converted via the vitB_12_-dependent enzyme methionine synthase to methionine. A third option is the PLP-independent reaction to S-adenosyl homocysteine (SAH) by SAH hydrolase (Figure 6). If vitB_6_ levels are low in the cell, homocysteine is primarily converted to SAH because the transulfuration pathway is affected, as well as tetrapyrrole biosynthesis, which is needed to make vitB_12_. As a consequence, SAH can accumulate, which inhibits S-adenosyl methionine (SAM)-dependent methylation reactions (Figure 6) [162]. This will cause a decrease in the production of certain neurotransmitters by reducing the synthesis of tetrahydrobiopterin, an essential co-factor for the hydroxylation of phenylalanine and tryptophan that serve as precursors for epinephrine, serotonin, and dopamine [140,163]. It is believed that this accumulation of either homocysteine or SAH, in connection with vitB_6_ deficiency, is an instigating cause for depressive symptoms and other mental health issues, as well as neurotoxic effects [138,140,154]. This notion is supported by animal studies where supplementation of vitB_6_ led to higher levels of serotonin in the brain [140].

The studies presented show a consistent pattern of lower dietary levels of vitB_6_ leading to higher levels of homocysteine, and decreased neurotransmitters in people that have confirmed mental health issues [138,140,153,154,164].

## 7. VitB_6_ Requirements in Human Nutrition

The sections above clearly emphasize the relevance of the vitamin for basic cellular metabolism in all organisms. It is, therefore, of interest to explore to what extent the vitamin is provided in our daily diet, and whether fortification with it may be recommendable. We have previously provided a review on vitB_6_ and human health [1], and will just provide a few current developments as an update.

The recommended dietary allowances (RDA) for vitB_6_ provided by the United States Food and Nutrition Board of the Institute of Medicine ranges between 1.3 mg (young adults) to 1.7 mg (adult males), and can reach up to 2 mg for lactating women. Taking these values into consideration, potato, for example, which is a staple food in many countries, represents an excellent nutritional source for this vitamin, as baked potatoes or potato chips contain up to 23% and 60% of the RDA values per 100 g product, respectively (Table 1). Many other products, such as bananas, nuts, and even meat or eggs, contain good to high amounts of the vitamin (Table 1). In addition, the vitamin is heat stable, and processing steps such as cooking or frying do not affect its content. Overall, in a healthy population that has access to a balanced diet, one can emphasize that vitB_6_ deficiency is uncommon. Although, certain age groups such as older adults (above age 45) have been reported to have significantly lower PLP plasma levels, and may benefit from a fortified vitB_6_ diet [165,166].

Deficiencies can be caused by high and continuous alcohol consumption and certain antiepileptic drugs, as well as severe renal diseases [167,168,169,170,171,172,173,174,175,176]. In addition, vitB_6_ deficiency can result from some malabsorption syndromes, such as Crohn’s disease, ulcerative colitis, or celiac disease, and certain genetic disorders such as homocystinuria may cause deficiencies in vitB_6_ [167,175,177,178,179].

If vitB_6_ deficiency occurs, it is often associated with dermatitis, microcytic anemia, or electroencephalographic abnormalities [167,175,180,181]. Sometimes, weakening of the immune function, convulsive seizures, and depression and confusion have also been reported [167,175,182,183,184,185,186].

In contrast, overdosage with the vitamin is hard to accomplish. The current daily tolerable upper intake level (UL) for adults (19 years and older) recommended by the Food and Nutrition Board of the Institute of Medicine in the United States is 100 mg [167]. Nevertheless, some case studies have been reported where high dosages of the vitamin caused neurological disorders [167,187,188,189]. It needs to be emphasized that often in these studies the daily levels exceeded the UL by far, and were provided over prolonged periods of time (for example 2000 to 6000 mg/day of pyridoxine for 2 to 40 months described by Schaumburg and co-workers [187]), making it not very likely that one would experience such symptoms under normal conditions [187,190,191,192].

## 8. VitB_6_ and the Potential to Develop New Medical Drugs

Knowledge of vitB_6_ and its related enzymes are used to treat diseases on three levels: supplementation of vitB_6_, targeting select enzymes in the vitB_6_ biosynthesis pathways, and targeting PLP-dependent enzymes. There are several reviews out that discuss this topic at length [164,193,194,195,196], so here we will only highlight a few interesting examples.

As vitB_6_ is important for neurotransmitter biosynthesis, one can anticipate that many neurological disorders such as Parkinson’s, Alzheimer’s, epilepsy, autism, and schizophrenia are affected by PLP availability [164]. In the case of pyridoxine-dependent epilepsy (PDE), recurrent neonatal or infantile seizures are alleviated by high doses of PLP [197]. There are currently no dosing recommendations, though the typical long-term doses of PLP prescribed do not exceed the UL [198]. PDE patients taking doses over 500 mg/day are closely monitored for adverse affects, such as sensory neuropathy [198]. PDE is caused by mutations in the ALDH7A1 (Antiquitin) gene in the lysine degradation pathway, which leads to the build-up of the lysine intermediate L-aminoadipate-semialdehyde (AASA) and its cyclic form, 1-piperideine-6-carboxylic acid (P6C) [199]. P6C can react with and thereby inactivate PLP. It is hypothesized that the subsequent depletion of PLP in the brain causes overexcitement and epileptic seizures due to reduced PLP-dependent biosynthesis of the inhibitory neurotransmitter γ-aminobutyric acid (GABA) [193]. Recently, a zebrafish model for PDE was created, by generation of an *aldh7a1* knockout line, that will allow researchers to better study this disease [200]. In another example, dopamine deficiencies are commonly responsible for the primary symptoms of Parkinson’s disease [164]. Looking at neurons of the *substantia nigra* region in the brain of humans, one of the four differentially expressed genes found was PDXK [164,201]. It is discussed that if PDXK is up-regulated in dopaminergic neurons, which are the main source of dopamine in the midbrain, this might alleviate some symptoms of Parkinson’s disease, although this particular method has not yet been explored [201]. However, other authors have cast doubts on these results because the studies were conducted in isolated populations with the possibility of a high degree of inbreeding due to marrying within a local community, along with the lack of the same differential expression of the four genes, including the pyridoxal kinase locus, in these populations [201,202,203].

In the remaining paragraphs, we will explore the role of pathogens and vitB_6_, a field that has been rapidly emerging, and that may provide interesting opportunities to develop new strategies to prevent infections.

VitB_6_ has already been known for quite some time to play a role in pathogen–host interactions. Some early work comes from the phytopathogen *Cercospora nicotianae*, a fungus that uses a secretable photosensitizer that generates ROS in response to illumination. This production of ROS is needed to facilitate penetration of host plant tissue with the fungal hyphae [204,205]. Intriguingly, a screen for *Cercospora* mutants sensitive to their own toxin resulted in the identification of fungi that were affected in vitB_6_ biosynthesis [206,207,208]. This was actually a first discovery that vitB_6_ is a very potent antioxidant that can efficiently protect against increasing ROS in the cell [207]. It was also a first discovery that it plays a critical role in the host–pathogen interplay.

Plants on the other hand also appear to take advantage of vitB_6_, but rather as a way to reduce their infection risk by pathogens. This notion is supported by a recent example from *Arabidopsis*, where mutants affected in de novo and *salvage* pathway genes displayed significantly higher susceptibility to infection by the bacterium *Pseudomonas syringae pv. tomato* (*Pst*) and the fungus *Botrytis cinerea* [209]. However, it remains open whether increased susceptibility of the plants was caused by a generally reduced metabolic rate, and thus lowered cellular fitness, or whether vitB_6_ is critical to induce an effective cellular defense response against a pathogen.

Another interesting example stems from the previously mentioned bacterium *P. luminescens* that symbiotically associates with the entomopathogenic nematode *H. bacteriophora* [210]. In the absence of this symbiotic relationship, *P. luminescens* is infectious to insects and other nematodes. A genetic screen for virulent deficient *P. luminescens* mutants found that reduced pathogenicity can be caused by a mutation in PdxB, an erythronate-4-phosphate dehydrogenase, which is part of the DXP-dependent de novo pathway [211]. *pdxB* mutants show an overall poor growth, which can be restored by supplementing growth media with PLP, but also with non-phosphorylated vitB_6_, demonstrating that the bacterium has efficient vitB_6_ uptake systems and a functional salvage pathway [211]. However, although the precise impact of vitB_6_ for pathogenicity is not clear at this point, a defined metabolic rate with sufficient PLP levels is discussed as a requirement to support either the mutualistic lifestyle or the ability to infect other organisms [211].

A correlation between vitB_6_ biosynthesis and pathogenicity has also been reported for three other bacteria, *Helicobacter pylori*, *S. pneumonia*, and *Mycobacterium tuberculosis* [22,212,213]. *H. pylori* is a flagellated bacterium that can cause stomach infections. Reduced activity in the vitB_6_ de novo synthesis pathway reduces the bacterium’s virulence and also impairs its ability to make glycosylated flagella, resulting in immobile bacteria cells [212]. *S. pneumoniae* is a Gram-positive bacterium that is responsible for a variety of severe infections, including pneumonia or meningitis. Loss in de novo biosynthesis genes did not fully prevent pneumonia infection in a mouse model, but it was able to produce significantly attenuated infection rates compared with wild type [22].

Similarly, loss of the de novo pathway in the tuberculosis-causing bacterium, *M. tuberculosis,* prevents the pathogen from persisting in host tissue [213]. Interestingly, apart from interfering with vitB_6_ biosynthesis directly in *M. tuberculosis*, it has also been found that the vitamin itself, taken as a supplement or added to a treatment, can improve the efficacy of tuberculosis treatments. For example, vitB_6_ (along with 11 other vitamins) was tested as a cofactor for *Mtb*LrpA, a transcriptional regulator that is thought to play an important role in the persistence of *M. tuberculosis*, and was found to inhibit MtbLrpA binding to DNA [214]. In other words, more vitB_6_ means more inhibition of MtbLrpA, which in turn causes reduced persistence of the bacterium. In another example, when the *M. tuberculosis pdx1* loss of function mutation was added to a tuberculosis vaccine background, as long as supplemental vitB_6_ was provided, the resulting vaccine was found to be safer for immunodeficient mice and profoundly more effective than the vaccine strain without mutated *pdx1* [215].

All of these examples led to the discussion of whether the vitB_6_ pathway could be used as a potential drug target to develop novel medications for preventing infections by the mentioned bacteria and other pathogens. The absence of the de novo vitB_6_ biosynthesis pathway in humans makes this a very promising approach; because in order for a potential drug to be effective, it needs to (1) be specific to the pathogen’s enzymes and metabolism without also targeting the host’s processes or causing undue damage to the host, and (2) interfere with or inhibit the pathogen’s metabolic process to such an extent that it cannot survive, reducing or eliminating the pathogen load so that the host can recover. A drug that targets PDX proteins and selective PLP-dependent enzymes that are necessary for pathogen survival can conceivably meet both of these requirements. In this context, we will mention malaria as a eukaryotic pathogen that may be a promising target for development of novel anti-malaria drugs utilizing the de novo vitB_6_ pathway.

Malaria, which, in severe cases, can cause death, is a devastating disease that continues to affect millions of people worldwide, especially around the equator. There were 214 million new cases of malaria and 438,000 deaths due to the disease in 2015 according to World Health Organization (WHO) estimates [216]. A major problem in the fight against malaria is the growing disease resistance of *Plasmodium falciparum* to the current anti-malarial drugs, requiring researchers to continually look for new drug targets. A 2014 review identified the PLP-dependent enzymes ornithine decarboxylase (ODX), the *P. falciparum* aspartate aminotransferase (AAT), and SHMT as potential targets, however, the corresponding host enzymes might also be affected, which must be taken into consideration [132,195]. Kronenburger and coworkers also suggested targeting PLP-related molecules in the Anopheles vector, such as 3-hydroxykynurenine transaminase (HKT), but more information on the structure of HKT is needed to be able to design a drug that targets it. We are not aware of any more recent work that sheds light on the structure of HKT or any drugs that have been developed specifically against vitB_6_ de novo enzymes. There may be other candidate targets to fight malaria that are currently more appealing to pursue, however, it is likely an oversight if these potential vitB_6_-related targets are abandoned as a tool to develop novel treatments against *P. falciparum* and other pathogens.

## 9. Conclusions

More than 80 years have passed since vitB_6_ was first described. Since then, we have come to understand how it is synthesized in the cell and its requirement in numerous biochemical reactions. There is also a growing amount of knowledge about the biological and physiological processes it affects across pro- and eukaryotic species. Yet there are still many aspects of the vitamin that need to be explored. Because it is required for so many processes, ranging from amino acid metabolism to stability of certain storage compounds or the biosynthesis of other vitamins, one would expect that its own biosynthesis and homeostasis is tightly controlled in the cell. However, regulatory aspects that control the homeostasis of vitB_6_ in the cell, on both the transcriptional and the post-translational level, are not well defined. Likewise, little knowledge has been generated about the cross-talk between the *salvage* and de novo pathways, and how catabolic processes may play a role in controlling vitB_6_ levels in the cell. We also have a poor understanding of how the biosynthesis of vitB_6_ is regulated in the metabolic network of a cell in context with environmental conditions, or in relation to developmental aspects. Another underexplored area is its subcellular and long-distance distribution and transport, which will be key to fully understanding the vitamin’s role in the cell.

The many ‘indirect’ aspects that appear to be affected by the vitamin, such as health, abiotic stress tolerance, pathogen susceptibility, or even the virulence of a pathogen, are very promising areas for vitB_6_ research. Investigations in these fields may lead to new findings and applications that result in novel treatments and medications to cope with stress, depression, or infections.

One interesting aspect to potentially explore deeper could be to use plants as primary producers of the vitamin to cope with potential deficiencies in the human diet, like with the ‘Golden Rice’ [217], where biofortification of rice plants with carotenoids was meant as a tool to improve the nutritional quality. Because UL levels are very high, it is unlikely that one would observe toxic side effects caused by such an approach. In fact, current literature shows that bioengineered plants with elevated vitB_6_ levels did not exceed, for example, 6 ng/mg fresh weight [26]. Rather, because vitB_6_ and other essential vitamins have such profound impacts on human health, and can be de novo synthesized by plants, phytonutrient biofortification could be a promising route in the future. In addition, beneficial impacts have also been described for the plants themselves, such as improved abiotic stress tolerance and increased seed size, and likely also affect pathogen tolerance, which are highly valuable traits for agriculture [26,209].

In conclusion, it will be interesting to see how the vitB_6_ field continues to develop, and what novel findings will add to the understanding and utilization of this important and ubiquitous vitamin.

## Figures and Tables

**Table 1:** Selected Foods and VitB_6_ Content based on the USDA Food Composition Database (https://ndb.nal.usda.gov).

Food Source	VitB_6_ [mg/100 g]
Crude Rice Bran	4.07
Vegetable Oil Spread (60% Fat)	3.75
Raw Garlic	1.235
Cooked Chicken Breast	1.157
Cooked Beef Liver	1.083
Roasted Pistachio Nuts	1.07
Cooked Yellow Fin Tuna	1.04
Top Round Boneless Steak	0.891
Cooked Sockeye Salmon	0.83
Potato Chips	0.8
Roasted Hazelnuts	0.62
Baked Potato	0.614
American Cheese	0.567
Flaxseeds	0.47
Feta Cheese	0.424
Raw Bananas	0.367
Raw Avocado	0.257
Hard-boiled Egg	0.121
American Cheddar	0.12
Dried Pine Nuts	0.09
Milk (2% Fat)	0.051

## Abbreviations

AASA	L-aminoadipate-semialdehyde
AAT	aspartate aminotransferase
ABC	ATP BINDING CASSETTE
ABRE	abscisic acid-responsive elements
ACC	1-aminocyclopropane-1-carboxilic acid
ALP	alkaline phosphatase
ALS	acetolactate synthase
BCAA	branched-chain amino acid
BCAT	branched-chain amino acid transaminase
BH4	tetrahydrobiopterin
CBS	cystathionine-β-synthase
DAP	deoxyxylose 5′-phosphate
DXP	deoxyxylose 5′-phosphate
ERE	ethylene-responsive elements
FMN	flavin mononucleotide
GAP	glyceraldehyde-3-phosphate
GLN	L-glutamine
GLU	L-glutamate
HKT	3-hydroxykynurenine transaminase
HTH	helix-turn-helix
MAT	methionine adenosyltransferase
MS	methionine synthase
MT	methyltransferase
MTHFR	methylenetetrahydrofolate reductase
NR	nitrate reductase
NUP1	NICOTINE UPTAKE PERMEASE 1
ODX	ornithine decarboxylase
P6C	1-piperideine-6-carboxylic acid
PDE	pyridoxine-dependent epilepsy
PDXA	Pyridoxine Biosynthesis Proteins A
PDXH	pyridoxine/pyridoxamine 5′-phosphate oxidase
PDXJ	pyridoxine 5′-phosphate synthase
PDXK	pyridoxal kinase
PHT	4-phosphohydroxy-L-threonine
PL	pyridoxal
PLK	pyridoxal kinase specific to PL
PLP	pyridoxal 5′-phosphate
PLR	pyridoxal reductase
PM	pyridoxamine
PMP	pyridoxamine 5′-phosphate
PN	pyridoxine
PNP	pyridoxine 5′-phosphate
PNPox	pyridoxine 5′-phosphate oxidase
PPL	pyridoxyllysine
PUP	purine permease
RDA	recommended dietary allowances
RIP	ribose-5-phosphate
ROS	reactive oxygen species
RUP	ribulose-5-phosphate
SAH	S-adenosylhomocysteine
SAHH	S-adenosylhomocysteine hydrolase
SAM	S-adenosylmethionine
SHMT	serine hydroxymethyltransferase
THF	tetrahydrofolate
UL	tolerable upper intake levels
vitB_12_	vitamin B_12_
vitB_6_	vitamin B_6_
